# Health-related quality of life and clinical outcomes following medial open wedge high tibial osteotomy: a prospective study

**DOI:** 10.1186/s12891-016-1076-x

**Published:** 2016-05-18

**Authors:** Christoph Ihle, Atesch Ateschrang, Leonard Grünwald, Ulrich Stöckle, Tim Saier, Steffen Schröter

**Affiliations:** BG Traumacenter Tübingen, Schnarrenbergstr. 95, 72076 Tübingen, Germany; BG Traumacenter Murnau, Prof.-Küntscher-Str. 8, Murnau, Germany

**Keywords:** Open wedge high tibial osteotomy, HTO, Varus deformity, Health-related quality of life, SF-36

## Abstract

**Background:**

Open wedge high tibial osteotomy (HTO) is an established method for the treatment of patients with varus malalignment and medial compartment osteoarthritis. In these patients, health-related quality of life (HRQL) can be improved by using this procedure. The purpose of the present study consisted in evaluating HRQL up to 18 months after HTO, comparing the results to values of the German normal population, and in analyzing the impact of preoperative HRQL on the postoperative clinical result. It was hypothesized that normal values in physical and mental health can be achieved within 18 months after operation. Study design: Prospective case series. Level of evidence: IV.

**Methods:**

120 patients were included in this prospective case series from 12/2008 to 12/2011. All patients underwent open wedge HTO without a bone graft using the TomoFix^TM^ plate. HRQL was assessed by using the SF-36 questionnaire, preoperatively, as well as 6, 12, and 18 months postoperatively. Regular scoring, norm-based scaling, and the physical and mental component summary scores (PCS and MCS) were evaluated. Clinical outcome was assessed by using Lequesne, Lysholm, HSS and IKDC Score.

**Results:**

HRQL could be described in 96 patients. The PCS of HRQL showed a statistically significant pre- to postoperative improvement (30.2 ± 13.4 to 45.9 ± 13.5 after 18 months). A reduced preoperative mental component summary score (MCS) resulted in lower values of each clinical score (*p* < 0.05) and in a prolonged duration of incapacity for work (MCS < 50:15.0 ± 12.8 weeks, vs. MCS ≥ 50: 9.1 ± 4.8 weeks, *p* < 0.05). MCS values improved from the pre- to postoperative measurements and comparable values to the normal population were reached already within 6 months after surgery (46.0 ± 14.9 pre-operatively and 48.5 ± 13.7 after 6 months), and after 18 months even a score of 49.5 ± 12.4 was achieved.

**Conclusion:**

Lower preoperative mental component score results in reduced postoperative clinical outcome and prolonged duration of incapacity for work after HTO. In contrast to PCS, MCS showed comparable values to the normal population within 6 months after HTO.

## Background

Open wedge high tibial osteotomy (open wedge HTO) is a widely used treatment option for patients with medial compartment osteoarthritis and varus malalignment. In the past, several studies have described a significant clinical and radiological improvement by using this established method [1, 2]. In most of the presented studies, well-known clinical scores, such as the KOOS (Knee Injury and Osteoarthritis Outcome Score), the International Knee Documentation Score (IKDC) or the Oxford Knee Score were used to describe the postoperative outcome [3–5]. Health-related quality of life (HRQL) became a popular tool to measure the clinical outcome after operative procedures in the field of orthopedic surgery as well [6–8]. In patients with full thickness rotator cuff tears, it could actually be demonstrated that the mental health of HRQL has a stronger association with patient-reported shoulder pain and function than tear size [9]. The SF-36 Questionnaire is an established and widely used tool for describing HRQL in various medical fields [10, 11]. In patients suffering from osteoarthritis of the knee joint, HRQL is significantly reduced, especially due to pain on the basis of cartilage damage [12].

Considering the general population, HRQL is decreasing with age [13]. To analyze the quality of life properly, a comparative interpretation regarding gender and age structure is necessary. Therefore, the norm-based scoring of the SF-36 Questionnaire, the mental component summary score (MCS), and the physical component summary score (PCS) can be used [14]. Although the summary scores can be interpreted more easily, the results should be thoroughly compared to the SF-36 scales before drawing conclusions [14, 15].

The great importance of preoperative psychological aspects on the postoperative clinical outcome has already been revealed in anterior cruciate ligament surgery [16]. So far, the precise influence of physical and mental components of quality of life on the postoperative clinical outcome after open wedge HTO is not known to the authors. No comparative interpretation on the values of the normal population regarding gender and age structure has become available so far. However, a therapeutic treatment of knee osteoarthritis requires a thorough understanding of the impact of such a treatment on the patient’s physical, social and psychological status [17]. HRQL, a measuring instrument for the outcome also considering mental aspects, has become popular in recent years, especially in evaluating treatment options for osteoarthritis of the knee [18–23]. Next to open wedge HTO, current surgical treatment options for osteoarthritis include unicompartmental knee arthroplasty (UKA) and total knee replacement (TKR) [24]. Recently, numerous studies have reported that psychological factors can influence the postoperative clinical outcome after TKR [25]. The mental status in primary TKR can affect the outcome and patient satisfaction [26].

An improvement in HRQL after open wedge HTO has been described several times, whereas randomized and controlled clinical trials comparing quality of life after open wedge HTO with other treatment options have not been available so far [27–29]. There does not exist any study comparing the results in quality of life with values of the normal population. Therefore, the purpose of the present study consisted in evaluating the HRQL after open wedge HTO without a bone graft using the TomoFix^TM^ plate, by comparing the results to values of the German normal population, and in analyzing the impact of the preoperative HRQL on the postoperative clinical result. 6 weeks after TKR, the values regarding MCS of HRQL decreased, before increasing again and plateauing at 1 year [30]. In the patients undergoing this surgery, the highest increase in PCS and MCS of HRQL was recorded between the first and the second year after surgery [30, 31]. Similarly to these results, we formulated our hypothesis that normal values in physical and mental health can be achieved within 18 months after operation.

## Methods

This prospective study was performed with the approval of the local ethics committee (University of Tübingen; 142/2008MPG2). Registration in the WHO register for clinical trials was carried out and written consent from the study participants was obtained. The focus of this project consisted in describing the health-related quality of life after an open wedge HTO and in determining the impact of the preoperative mental situation on the postoperative clinical result. HRQL was compared to available values of the German normal population and differences between both groups were evaluated. Therefore, patients with symptomatic varus malalignment and medial compartment osteoarthritis who received an open wedge HTO between December 2008 and December 2011 using the TomoFix^TM^ plate (DePuySynthes, Solothurn, Switzerland) were consecutively included in this study. Exclusion criteria were previous or acute infections of the knee joint, clinically symptomatic and diagnosed osteoporosis or being under 18 years of age. Therefore, 120 patients were eligible for this study, and were consecutively evaluated by clinical and radiological examination at the following predefined time points of interest: preoperative, 6, 12 and 18 months after surgery. In order to obtain the most homogenous population possible, all patients who received additional procedures, which could have had an influence on the results of HRQL, during surgery or during the follow-up period, were excluded from data analysis. Cartilage repair (microfracture, autologous cartilage transplantation) and ligament reconstructions were defined as additional procedures. Patients who suffered from complications during the follow-up period were also excluded from the study. In detail, these complications include postoperative wound and implant infections, delayed bone healing, the need for a follow-up operation and implant failure. For clinical evaluation, the following established and widespread knee surgery scores were used: the Lequesne Score [32], the Lysholm-Score [33, 34], the HSS-Score [35], and the International Knee Documentation Score (IKDC) [36–38]. In addition, the duration of incapacity for work was described. All clinical and radiological examinations were performed by a single examiner.

### Health-related Quality of Life (HRQL)

HRQL was recorded by using an established measurement method, i.e. the SF-36 questionnaire [10, 39] (German version) preoperatively, as well as at 6, 12, and 18 months follow-up. The reliability of the SF-36 questionnaire across diverse patient groups was tested and a good to excellent reliability coefficient of 0.85 was found [40]. Psychometric validation of the German SF-36 achieved comparable results for data completeness, reliability, and construct validity with other European samples [41–43].

The questionnaire consists of 36 questions. In the present study, a time period of 4 weeks before the assessment takes place is considered and the interview form was used. With the values of these 36 questions, eight scales describing physical and mental health can be assessed. While Physical Functioning (PF), Role Physical (RP), Bodily Pain (BP), and General Health represent the physical part, Vitality (VT), Social Functioning (SF), Role Emotional (RE), and Mental Health (MH) describe the mental aspect of HRQL. Firstly, regular scoring (RS, 0–100 points) was used. However, due to poorer general health in older people, HRQL is decreasing with age, so that regular scoring cannot be used for a comparison of the obtained values to values of the normal population. Therefore, in order to compare each scale to the values of the normal population irrespective of age dependent differences, a transformation to norm-based scoring (NBS) was essential. Based on NBS as described by Ware [43], a comparative interpretation regarding gender and age structure is possible. While an NBS value of below 50 for study participants is below the average of the respective normal sample, a score of over 50 is above this average. In order to allow for an interpretation of the results in a simpler and standardized way, a transformation of the values into a physical and mental component summary score (PCS and MCS) is widely used and was also performed in this study.

### Preoperative planning and surgical technique

Preoperatively, a deformity analysis and digital planning were conducted in all patients with the digital planning software mediCAD (Hectec, Landshut, Germany). Surgery was performed under spinal or general anesthesia. Single-shot antibiotics and prophylactic low-dose heparin were used. To secure the indication for the procedure of a high tibial osteotomy, we performed an arthroscopy in each patient as a first step. However, in some cases, we performed an arthroscopy in advance as an outpatient surgery. Correction of the varus alignment was achieved by using either an infrared-based navigation tool (OrthoPilot®, Aesculap, BBraun, Tuttlingen, Germany), or by creating the preoperatively calculated wedge base height. Overall, the aim of correction was an overcorrection of 2–3° of valgus. The open wedge HTO surgical technique consisted of a biplanar 130° L-shaped osteotomy without a bone graft or bone substitute, as described by Staubli et al. [44] as well as by Lobenhoffer and Agneskirchner [45]. Briefly, a 4–6 cm longitudinal incision was made 5 cm distal to the knee joint line (orientation was distal to the pes anserinus). From the medial side, 2 k-wires were positioned parallel to the tibial slope above the pes anserinus, targeting the tip of the tibiofibular joint, under fluoroscopic control. The transverse osteotomy bone cut was made up to 1 cm to the lateral cortical bone; for the ascending osteotomy, the tibial tuberosity bone was cut completely at an angle of 130°. The osteotomy was slowly spread to achieve a gap size according to the preoperative digital planning or navigation tool. The TomoFix plate was placed anteromedially and fixed proximally with four 5.0 mm locking screws. To compress the lateral hinge, a bicortical temporary lag screw was inserted in the first plate hole distal to the osteotomy. In the remaining distal holes, 5.0 mm monocortical locking screws were inserted. Finally, the bicortical screw was removed and a bicortical locking screw was inserted. The tissue layers were closed, and a drain was placed.

### Postoperative rehabilitation

Postoperatively, patients were assigned either to 20 kg partial weight-bearing using two crutches for 11 days or to 6 weeks of partial weight-bearing followed by full weight-bearing. During the study, both aftercare protocols were used in our hospital. No braces or casts were used. Active physiotherapy was started postoperatively after the removal of the drain. In addition, the patients used an active motion splint (CAMOped; OPED, Valley/Oberlaindern, Germany) for 6 weeks. The active motion splint is a kind of "bed bike", like a continuous passive motion machine that is used for active therapy rather than for fixation or extra stability.

### Statistical analysis

SPSS 21 for Windows and GraphPad Prism for Mac (GraphPad Software, Version 5) were used for statistical analysis and graphical representation respectively. For statistical analysis, one-way analysis of variance or analysis of variance performed with repeated measures were used. Subsequent pairwise comparisons of analysis of variance with repeated measures were analyzed by alpha-adjusted Bonferroni corrections, to check if possible significant results of the repeated measures ANOVA proved to lead to significant differences in pairwise comparisons. Furthermore dependent or independent t-tests were used according to the data. *P* < 0.05 was considered to be statistically significant.

## Results

### Patient characteristics

**Fig. 1:**
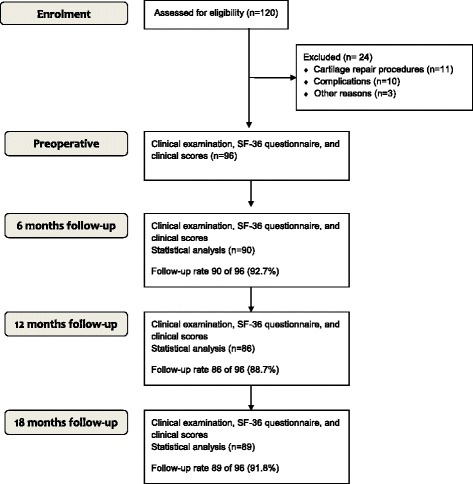
CONSORT Patient flow-diagram

Prospectively, 120 patients were included in this study, before receiving an open wedge HTO (Fig. 1). After exclusion of 24 patients, 96 participants were used for further statistical evaluation with an average age of 46 ± 8 years (71.9 % male, 28.1 % female). However, all patients who had received additional procedures with a probable influence on the quality of life were excluded from the data analysis, so that all cartilage repair procedures (microfracture, autologous cartilage transplantation, *n* = 11) were eliminated from this study. Two more participants were excluded from the data analysis: One of them had a PCL lesion, while the other required a PCL reconstruction during the follow-up period. Another patient had a fracture of the medial condyle and the HTO was used as a salvage procedure with the aim of delaying arthroplasty. 6 months later however, this patient required an arthroplasty, and was therefore excluded from our study as well. In *n* = 10 cases (8 %), complications were observed: One patient exhibited delayed bone healing, and therefore a bone graft transplantation from the iliac crest was performed. Two other participants received early bone graft transplantation after 4 months, because they suffered from pain around the osteotomy, which made the early removal of the implant possible. In three cases, we observed an infection: Two patients had subcutaneous infections, while the third one suffered from a deep infection, so that the implant had to be removed after 13 days, and fixation of the osteotomy had to be performed with a fixator in order to maintain the correction. Furthermore, bone transplantation with harvesting from the iliac crest was required after the infection had healed. After complete bone healing, the patient was satisfied with the results. In two cases, implant failure occurred: One of these patients suffered a trauma with a twisting of the knee, while the other had no trauma, so that no reason for the failure could be found. In the follow-up, we observed one case with overcorrection and one with undercorrection, who both underwent successful revision surgery for recorrection. In total, *n* = 24 (20 %) patients were excluded from further statistical analysis. Patient characteristics are presented in Table 1.

**Table 1:** Characteristics of patients

	Total
Number of included patients	*n* = 96 (120)
Age	46 ± 8 years
Gender	71.9 % male, 28.1 % female
Body Mass Index (BMI)	28.8 ± 4.7
Affected side	*n* = 43 left, *n* = 54 right
Duration of incapacity for work	11.7 ± 9.4 weeks (*n* = 73)

### Health-related quality of life (SF-36 questionnaire)

**Fig. 2:**
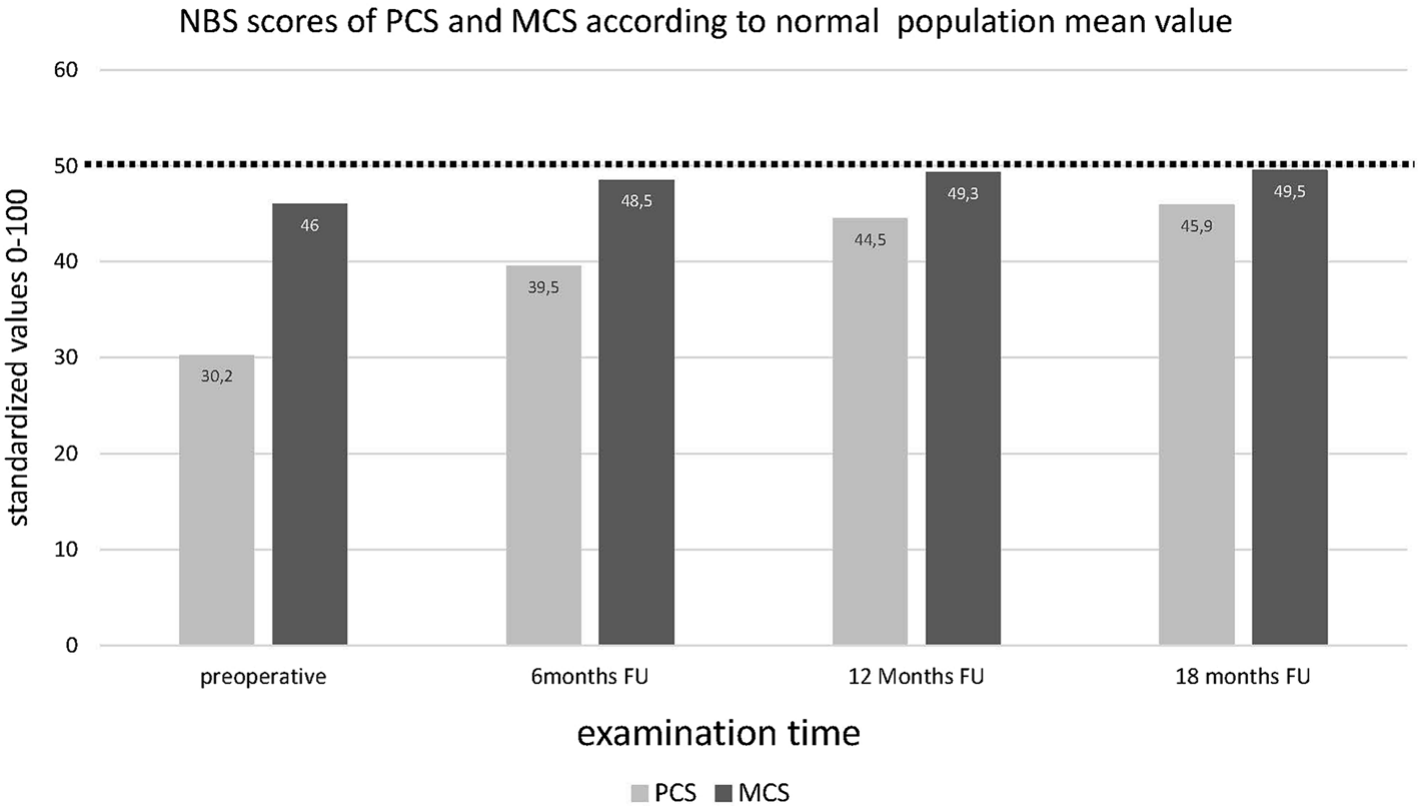
Norm-based scoring of the physical component summary score (PCS) and the mental component summary score (MCS) of health-related quality of life (SF-36) [mean values included in the bars; SD is illustrated in Table 2] compared to the mean value of the German normal population (NP) – indicated by the dotted line (M = 50). A significant clinical approximation of MCS in all postoperative results comparable to the German normal population was found

The results of HRQL (Regular scoring and NBS) at all predefined examinations are presented in Table 2. Repeated measures ANOVA (factor time – four steps) was performed with NBS scores. All scales, as well as the Physical Component Score (PCS), indicated significant improvements over time. The results and statistical values are included in Table 2. In order to check if these significant initial results of the repeated measures ANOVAs resulted in significant differences between examination times, we conducted pairwise comparisons with alpha-adjusted Bonferroni corrections. These pairwise comparisons are described in Table 3. The physical and mental components of HRQL (SF-36 questionnaire) showed clinically significant reduced preoperative values in comparison to the normal population (Fig. 2). Although the mental component summary score (MCS) did not exhibit statistically significant changes over time, the comparison of the postoperative values to the normal population showed clinically significant postoperative values comparable to the normal population at each follow-up examination (Fig. 2). Median duration of incapacity for work was 11.7 ± 9.4 weeks (*N* = 73). To compare the impact of preoperative mental status in HRQL, we divided our study sample into two groups (1) high preoperative MCS and (2) low preoperative MCS. Subgroups were divided by the mean value of the standardized value (=50). Our results indicated a strong correlation between MCS and the conducted clinical scores (Table 4), as the performed ANOVA revealed significant differences for all conducted comparisons between groups. In order to analyze the influence of possible co-founders, such as the use of a navigation system and the different postoperative rehabilitation protocols, the differences between the preoperative assessment at 6 months and at 18 months of follow-up were calculated. The differences between the groups of 11 days with 20 kg partial weight-bearing and of 6 weeks with 20 kg partial weight-bearing as well as between the use of intraoperative navigation and of preoperative digital planning regarding PCS and MCS are illustrated in Table 5. The only statistically significant difference was found in the age variable (age > 40 and age ≤ 40, respectively) which exhibited a significant difference of *p* < .05. However, as the sizes of both samples were fairly incomparable for this variable (*N* = 72 vs. *N* = 14), we do not want to draw too much attention to this finding, but would rather like to point out that age differences should be kept in mind as an aspect worth analyzing in further studies.

**Table 2:** SF-36 results: ANOVA to describe changes over time for regular scoring (0–100) and norm-based scaling, each

	(1) Preoperative M (SD)	(2) 6 months M (SD)	(3) 12 months M (SD)	(4) 18 months M (SD)	F	Df	*p*	η^2^
PF (Physical Functioning)								
NBS RS	27.4 (±16.9) 52.4 (±25.9)	35.9 (±17.1) 66.8 (±25.0)	42.4 (±14.6) 76.7 (±22.7)	44.7 (±13.9) 80.5 (±21.3)	37.452	3	<0.001**	0.327
RP (Role Physical)								
NBS RS	33.0 (±15.3) 38.0 (±43.2)	41.6 (±14.5) 61.7 (±43.1)	44.9 (±14.2) 72.1 (±40.1)	46.3 (±13.9) 75.3 (±41.0)	22.109	2.571	<0.001**	0.223
BP (Bodily Pain)								
NBS RS	34.5 (±8.2) 38.4 (±21.1)	41.0 (±9.4) 58.1 (±24.5)	45.6 (±9.3) 67.9 (±24.8)	46.5 (±10.5) 70.0 (±28.4)	46.748	2.843	<0.001**	0.378
GH (General Health)								
NBS RS	46.6 (±10.9) 60.8 (±20.2)	50.6 (±10.2) 68.3 (±19.8)	51.7 (±10.6) 70.8 (±19.3)	50.8 (±11.9) 68.8 (±22.8)	10.469	2.906	<0.001**	0.121
VT (Vitality)								
NBS RS	43.0 (±11.7) 52.7 (±19.7)	47.1 (±11.3) 59.6 (±18.9)	48.9 (±11.3) 62.6 (±18.8)	49.1 (±12.0) 63.0 (±20.5)	10.093	2.764	<0.001**	0.117
SF (Social Functioning)								
NBS RS	41.6 (±13.1) 74.8 (±22.8)	45.6 (±12.8) 81.8 (±21.7)	46.4 (±13.4) 83.2 (±23.4)	46.9 (±12.2) 83.9 (±21.7)	6.093	2.865	0.001**	0.074
RE (Role Emotional)								
NBS RS	38.8 (±19.1) 64.2 (±46.4)	43.8 (±16.0) 75.9 (±39.9)	46.3 (±14.5) 82.2 (±35.7)	47.6 (±13.9) 85.4 (±34.1)	10.612	2.178	<0.001**	0.121
MH (Mental Health)								
NBS RS	46.4 (±12.8) 68.6 (±20.1)	48.3 (±12.0) 71.7 (±18.6)	50.5 (±11.2) 75.1 (±17.7)	50.4 (±11.9) 75.1 (±18.7)	6.002	2.812	.001**	0.073
PCS (Physical Component Summary Score)								
NBS RS	30.2 (±13.4) –	39.5 (±13.5) –	44.5 (±12.9) –	45.9 (±13.5) –	40.993	2.368	<0.001**	0.350
MCS (Mental Component Summary Score								
NBS RS	46.0 (±14.9) –	48.5 (±13.7) –	49.3 (±12.6) –	49.5 (±12.4) –	3.761	2.543	0.017*	0.047

*NBS* Norm-Based Scaling, *RS* Regular Scoring; Df corrected according to Levene-Test of Significance, if necessary

**Table 3:** SF-36 results: paired comparisons of SF-36 scales – effects of time

Pairwise Comparison (Bonferroni)	Time	Average distance	*p*
PF (Physical Functioning)	(1) vs. (2)	−8.049	0.002**
	(1) vs. (3) (1) vs. (4)	−14.749 −16.935	<0.001** <0.001**
	(2) vs. (3) (2) vs. (4) (3) vs. (4)	−6.700 −8.886 −2.186	<0.001** <0.001** 0.262
RP (Role Physical)	(1) vs. (2)	−8.873	<0.001**
	(1) vs. (3) (1) vs. (4)	−12.107 −12.746	<0.001** <0.001**
	(2) vs. (3) (2) vs. (4) (3) vs. (4)	−3.234 −3.872 −0.639	0.272 0.068 n.s.
BP (Bodily Pain)	(1) vs. (2) (1) vs. (3) (1) vs. (4) (2) vs. (3) (2) vs. (4) (3) vs. (4)	−7.174 −10.591 −11.618 −3.417 −4.444 −1.027	<0.001** <0.001** <0.001** 0.005* 0.002* n.s.
GH (General Health)	(1) vs. (2) (1) vs. (3) (1) vs. (4) (2) vs. (3) (2) vs. (4) (3) vs. (4)	−4.070 −5.453 −3.994 −1.383 0.076 1.459	0.001** <0.001** 0.002* 0.915 n.s. 0.836
VT (Vitality)	(1) vs. (2) (1) vs. (3) (1) vs. (4) (2) vs. (3) (2) vs. (4) (3) vs. (4)	−3.589 −5.560 −5.630 −1.971 −2.040 −0.070	0.013* <0.001** <0.001** 0.643 0.439 n.s.
SF (Social Functioning)	(1) vs. (2) (1) vs. (3) (1) vs. (4) (2) vs. (3) (2) vs. (4) (3) vs. (4)	−3.602 −5.511 −5.262 −1.908 −1.660 0.248	0.120 0.004* 0.007* n.s. n.s. n.s.
RE (Role Emotional)	(1) vs. (2) (1) vs. (3) (1) vs. (4) (2) vs. (3) (2) vs. (4) (3) vs. (4)	−5.708 −8.608 −9.539 −2.900 −3.832 −0.932	0.054 0.002* <0.001** 0.536 0.012* n.s.
MH (Mental Health)	(1) vs. (2) (1) vs. (3) (1) vs. (4) (2) vs. (3) (2) vs. (4) (3) vs. (4)	−1.980 −4.029 −4.458 −2.049 −2.477 −0.428	0.553 0.017* 0.005* 0.496 0.187 n.s.
PCS (Physical Component Summary Score)	(1) vs. (2) (1) vs. (3) (1) vs. (4) (2) vs. (3) (2) vs. (4) (3) vs. (4)	−8.942 −13.856 −14.875 −4.915 −5.933 −1.018	<0.001** <0.001** <0.001** <0.001** <0.001** n.s.
MCS (Mental Component Summary Score	(1) vs. (2) (1) vs. (3) (1) vs. (4) (2) vs. (3) (2) vs. (4) (3) vs. (4)	−2.665 −4.025 −3.882 −1.360 −1.216 0.144	0.360 0.079 0.065 n.s. n.s. n.s.

(1) = preoperative, (2) = 6 months, (3) = 12 months, (4) = 18 months

**Table 4:** SF-36 results: Connection of the preoperative quality of life (MCS) to the postoperative values of the clinical scores (18 Months FU), and to the duration of incapacity for work (weeks)

Clinical Score (FU 18 months)	(1) Preoperative MCS < 50	(2) Preoperative MCS ≥ 50	T	df	*p*
IKDC	63.2 ± 20.4 (*N* = 44)	81.5 ± 13.9 (*N* = 41)	−4.778	83	<.001**
HSS	86.1 ± 14.8 (*N* = 44)	94.1 ± 10.3 (*N* = 42)	−2.904	84	.005*
Lysholm	77.6 ± 22.7 (*N* = 44)	89.7 ± 15.8 (*N* = 42)	−2.854	84	.005*
Lequesne	5.1 ± 4.8 (*N* = 43)	1.6 ± 2.9 (*N* = 42)	4.083	83	<.001**
Duration of incapacity for work	15.0 ± 12.8 (*N* = 39)	9.1 ± 4.8 (*N* = 39)	2.680	68	0.009*

**Table 5:** Differences of 18 months vs. preoperative and of 6 months vs. preoperative: Comparison of the different subgroups

	Difference 18 months-preop			Difference 18 months-preop			Difference 18 months-preop		
	11 days	6 weeks	*p*-value	No Navigation	Navigation	*p*-value	Age > 40	Age ≤ 40	*p*-value
PCS	16.3 ± 15.0	14.1 ± 13.9	n.s.	14.8 ± 14.3	15.8 ± 14.9	n.s.	14.0 ± 13.4	21.7 ± 18.4	*p* < 0.05*
MCS	4.8 ± 12.7	1.9 ± 16.4	n.s.	1.6 ± 16.0	5.4 ± 12.8	n.s.	4.0 ± 13.0	2.6 ± 16.6	n.s.
	Difference 6 months-preop			Difference 6 months-preop			Difference 6 months-preop		
	11 days	6 weeks	*p*-value	No Navigation	Navigation	*p*-value	Age > 40	Age ≤ 40	*p*-value
PCS	10.2 ± 14.2	8.0 ± 2.3	n.s.	11.4 ± 12.7	7.0 ± 16.3	n.s.	8.3 ± 13.4	13.3 ± 3.8	n.s.
MCS	2.9 ± 13.3	1.0 ± 10.1	n.s.	2.3 ± 10.9	1.7 ± 13.3	n.s.	2.7 ± 12.3	−1.4 ± 11.1	n.s.

## Discussion

The most important finding of the present study was that patients with lower preoperative mental component scores experienced a statistically significantly reduced postoperative clinical outcome and a prolonged duration of incapacity for work after open wedge HTO. Moreover, in contrast to the physical component score, the mental component score exhibited values that were comparable to the normal population already 6 months after surgery. Considering the stated hypothesis, norm values in mental health can be achieved within 18 months after operation, whereas physical health is still restricted.

Reports of health-related quality of life after open wedge HTO are very limited, so that there still exists a lack of data concerning this topic. In the present study, the health-related quality of life in open wedge HTO was described in a prospective way by using the SF-36 questionnaire. Furthermore, the impact of the HRQL on the clinical outcome was analyzed.

Although the physical aspect in health-related quality of life was even further reduced in comparison to the mental components, in the preoperative period, both values were significantly lower in our cohort of patients with medial compartment osteoarthritis than the values of the normal population. Surprisingly, the preoperative values of the physical component score were inferior to the described preoperative status of patients who were undergoing elective total knee arthroplasty in the United States [46]. Certainly, although norm-based values are different between the countries, the populations of Germany and USA are nevertheless comparable [47]. Picavet et al. evaluated the health-related quality of life by using the SF-36 questionnaire in 3664 Dutch patients with musculoskeletal diseases. One of the poorest HRQL results could be found in patients with osteoarthritis of the lower extremity [12]. These results are comparable to other studies evaluating the quality of life in patients with osteoarthritis of the lower extremity, which strengthens the importance of using quality of life as an outcome measurement tool for evaluating the treatment result after open wedge HTO [12, 27]. Our data showed a pre- to postoperative improvement in all scales of the SF-36 questionnaire. This is comparable to the available literature. McNamara et al. studied 138 patients for 24 months after open wedge HTO. The SF-12 questionnaire was used to describe health-related quality of life and significant pre- to postoperative improvements were reported [27]. In our data, for the absolute terms of PCS as well as of MCS, the largest increase was reported between the preoperative and the 6 months follow-up, while there were only small improvements in the period from 12 to 18 months after surgery. For the MCS, similar values in comparison to the German normal population were achieved already 6 months after the surgery. Although there are significant restrictions compared to the normal population regarding physical status up to 18 months postoperatively, the PCS was still increasing at the last follow-up. Therefore, a further improvement in PCS can be expected. Maffulli et al. indicated significant pre- to postoperative results in all SF-36 scales 2 years after operation [28]. The restriction compared to the normal population at 18 months after surgery is not unexpected regarding the dimensions of the PCS. Besides general physical components of the quality of life, the Physical Functioning, Role Physical, and Bodily Pain scales contain components describing clinical results that are also used in regular scores describing a disease-specific clinical outcome. With regular scoring, a direct comparison to the values of the normal population regarding gender and age structure is not possible. Despite the significant pre- to postoperative improvements, the postoperative results of regular scoring also indicate restrictions compared to healthy people.

The finding that the preoperative mental status of the patient has an influence on the postoperative result should be taken into account in preoperative planning and in the use of this procedure. Feucht et al. demonstrated the influence of the preoperative expectations of the patient on the postoperative clinical outcome in patients undergoing cruciate ligament surgery [16]. These findings strengthen our results and the importance of considering preoperative psychological components in the preoperative planning of open wedge HTO. In patients with a reduced preoperative mental status in comparison to the values of the normal population, a reduced postoperative clinical outcome and a prolonged duration of incapacity for work can be expected. An incapacity for work of 3 months after open wedge HTO and the impact of the work load according the REFA classification have already been described [48]. Faschingbauer et al. reported a comparable duration of 16.7 ± 15.6 weeks of the return to work after HTO [49]. The influence of the preoperative mental status on the incapacity for work is unknown. In the present study, the incapacity for work was 15.0 ± 12.8 weeks, if the MCS was <50 and 9.1 ± 4.8 weeks, if the MCS was ≥50. This knowledge is completely novel. Bode et al. presented comparable results after high tibial osteotomy combined with autologous chondrocyte implantation [1]. In contrast, patients receiving additional procedures have been excluded from our study, as additional procedures could influence the health-related quality of life after open wedge HTO. Therefore, to the knowledge of the authors, the present study is the first to describe the quality of life after open wedge HTO without a bone graft comparing the results to the values of the German normal population. Indeed, long-term data on the quality of life after open wedge HTO compared to values of the normal population are still missing.

There are some limitations to this study, however: Firstly, the degree of osteoarthritis and its influence on the HRQL were not assessed. Several studies reported significantly statistically reduced values in health-related quality of life in osteoarthritis patients [12], whereas Pang et al. presented no correlation between the radiological degree of osteoarthritis in 466 osteoarthritis patients by using the widespread Kellgren Lawrence Classification, and the health-related quality of life using the SF-36 questionnaire [50]. In patients with osteoarthritis, pain, as a symptom of OA, has the greatest impact on HRQL. Considering the SF-36 Questionnaire, Bodily Pain is a part of the Physical Component Summary Score (PCS) and should not influence MCS values. Furthermore, we did not determine, if any of the patients had a psychiatric disease that could influence the results regarding health-related quality of life.

## Conclusions

A lower preoperative mental component score results in a significantly reduced postoperative clinical outcome and in a prolonged duration of incapacity for work after open wedge HTO. Moreover, in contrast to the physical component score, the mental component score exhibited comparable values to the normal population already 6 months after open wedge HTO.

## Ethics approval and consent to participate

This prospective study was performed with the approval of the local ethics committee (University of Tübingen; 142/2008MPG2). Registration in the WHO register for clinical trials was carried out and written consent from the study participants was obtained. A registration in the German register for clinical trials (approved primary register in the WHO network) is present (DRKS00005614). All procedures performed in this study were in accordance with the ethical standards of the institutional research committee and with the 1964 Helsinki Declaration and its later amendments or comparable ethical standards.

## Consent for publication

“Not applicable”.

## Availability of data and materials

The ‬dataset ‬supporting ‬the ‬conclusions ‬of ‬this ‬article ‬is ‬not‬ available ‬in ‬an ‬open ‬access ‬repository ‬because ‬it ‬is ‬part ‬of an ‬institutional ‬dataset ‬that ‬is ‬still ‬under ‬use. ‬If ‬there ‬is interest ‬in ‬exploring ‬specific ‬issues, ‬please ‬contact ‬the‬ corresponding ‬author ‬(CI).

## Abbreviations

HRQL: Health-related quality of life
HTO: Open wedge high tibial osteotomy
MCS: Mental component summary score
PCS: Physical component summary score
